# Pull-down combined with proteomic strategy reveals functional diversity of synaptotagmin I

**DOI:** 10.7717/peerj.2973

**Published:** 2017-02-08

**Authors:** Tianyao Guo, Zhigui Duan, Jia Chen, Chunliang Xie, Ying Wang, Ping Chen, Xianchun Wang

**Affiliations:** Key Laboratory of Protein Chemistry and Developmental Biology of Ministry of Education, College of Life Sciences, Hunan Normal University, Changsha, Hunan, P. R. of China

**Keywords:** Function, Diversity, C2 domain, Interaction protein, Synaptotagmin I

## Abstract

Synaptotagmin I (Syt I) is most abundant in the brain and is involved in multiple cellular processes. Its two C2 domains, C2A and C2B, are the main functional regions. Our present study employed a pull-down combined with proteomic strategy to identify the C2 domain-interacting proteins to comprehensively understand the biological roles of the C2 domains and thus the functional diversity of Syt I. A total of 135 non-redundant proteins interacting with the C2 domains of Syt I were identified. Out of them, 32 and 64 proteins only bound to C2A or C2B domains, respectively, and 39 proteins bound to both of them. Compared with C2A, C2B could bind to many more proteins particularly those involved in synaptic transmission and metabolic regulation. Functional analysis indicated that Syt I may exert impacts by interacting with other proteins on multiple cellular processes, including vesicular membrane trafficking, synaptic transmission, metabolic regulation, catalysis, transmembrane transport and structure formation, etc. These results demonstrate that the functional diversity of Syt I is higher than previously expected, that its two domains may mediate the same and different cellular processes cooperatively or independently, and that C2B domain may play even more important roles than C2A in the functioning of Syt I. This work not only further deepened our understanding of the functional diversity of Syt I and the functional differences between its two C2 domains, but also provided important clues for the further related researches.

## Introduction

Synaptotagmins represent a family of membrane proteins consisting of more than ten isoforms expressed in brain and other organs (Craxton, 2007; Chapman, 2008). Many studies on the structure, biochemical/biophysical properties and functions of synaptotagmins indicate that they play important roles in exocytosis, endocytosis and some other cellular processes (Geppert et al., 1994; Tucker & Chapman, 2002; Dai et al., 2004; Dai et al., 2007; Martens, Kozlov & McMahon, 2007; Chapman, 2008; Gustavsson & Han, 2009; Xu et al., 2013; Bacaj et al., 2015; Xu et al., 2014; Pérez-Lara & Jahn, 2015). Synaptotagmin I (Syt I) is the best characterized form of synaptotagmin and is most abundant in the brain (Stein et al., 2007; Chapman, 2008; Chapman et al., 1996; De Wit et al., 2009; Lee et al., 2010; Xue et al., 2010; Van den Bogaart et al., 2011; Vrljic et al., 2010; Vrljic et al., 2011; Yao et al., 2011; Vennekate et al., 2012; Seven et al., 2013; Zhou, Brewer & Rizo, 2013; Xie et al., 2014; Inoue et al., 2015; Bai et al., 2016; Zhou et al., 2015; Park et al., 2015; Pérez-Lara et al., 2016; Lyubimov et al., 2016). The primary structure of Syt I consists of a single N-terminal membrane-spanning domain, a short intracellular domain, and a large cytoplasmic domain consisting of tandem C2 domains, C2A and C2B, connected by a linker (Perin et al., 1991). Acting as a major Ca^2+^ sensor, Syt I plays important roles in neurotransmitter release and many other cellular processes (Ernst & Brunger, 2003; De Wit et al., 2009; Inoue et al., 2015; Bai et al., 2016; Kuzuya et al., 2016). The C2 domains of Syt I are the main functional regions and can automatically fold into the binding modules for Ca^2+^ , proteins and phospholipids, etc. (Geppert et al., 1994; Shao et al., 1996; Shao et al., 1997; Shao et al., 1998; Ubach et al., 1998; Ubach et al., 2001; Fernandez et al., 2001; Tucker & Chapman, 2002; Araç et al., 2006; Xue et al., 2008; Yoshihara, Guan & JT, 2010; Kuzuya et al., 2016). Recent observation suggests that the intrinsically disordered region between Syt I’s transmembrane helix and C2A is a key route for communication of lipid organization to the adjacent C2 domains (Fealey et al., 2016) . Through the use of site-directed fluorescent probes, it was found that in response to Ca^2+^ the Ca^2+^ -binding loops of C2A partially insert into phosphatidylserine (PS)/phosphatidylcholine (PC) bilayers (Chapman & Davis, 1998). The PS-binding activity of C2B is markedly enhanced by the presence of the adjacent C2A domain, demonstrating that C2A and C2B cooperate to bind to membrane (Bai, Wang & Chapman, 2002). Fluorescence resonance energy transfer studies conducted on Syt I demonstrated that Ca^2+^ concentrations required for membrane fusion induced a conformational change that brings the two Ca^2+^ -binding C2 domains in Syt I closer together, suggesting a mechanism for Syt I function at the presynaptic plasma membrane that involves the self-association of C2 domains (García, Forde & Godwin, 2000). The accumulated experimental evidences demonstrate that the two C2 domains may play synergetic roles in the functioning of Syt I and, comparatively, C2B domain seems to play even more important roles with the help of other molecules including proteins and non-proteinaceous molecules (Robinson, 2015; Kedar et al., 2015; Brewer et al., 2015).

As a calcium-phospholipid binding protein, Syt I is essential for synchronous synaptic vesicle fusion and exocytosis, whereas membrane fusion itself relies on the three SNARE proteins: synaptobrevin on the vesicle membrane, and syntaxin and SNAP-25 on the target plasma membrane (Geppert et al., 1994; Otto, Hanson & Jahn, 1997; Chapman, 2008; Holt et al., 2008; Van den Bogaart et al., 2010; Van den Bogaart & Jahn, 2011; Hernandez et al., 2012; Zhou et al., 2015). Syt I was shown to regulate the process of exocytosis by interacting with SNAP-25 (Mohrmann et al., 2013) and the syntaxin/SNAP-25 dimer (Rickman et al., 2004). Our previous work also demonstrated that syntaxin can bind to the C2B domain (Xie et al., 2014). Some other proteins participate in the regulation of synaptic vesicle exocytosis by interacting with Syt I, such as Dishevelled-1 (Ciani et al., 2015), APP (Gautam et al., 2015), tubulin (Honda et al., 2002), VCP (valosin-containing protein) (Sugita & Südhof, 2000), *β* -SNAP (soluble NSF attachment protein) (Schiavo et al., 1995), clathrin AP-2 (Zhang et al., 1994), *α* -latrotoxin receptor (Petrenko et al., 1991), etc.

Until now, although some proteins binding to the C2 domains of Syt I were identified and characterized, there has not been a comprehensive analysis of the proteins interacting with the C2 domains. In the present study, the pull-down combined with proteomic strategy was employed to identify and comparatively analyze the proteins interacting with the C2 domains of Syt I in order to further understand the C2 domain-interacting proteins and thus the functional diversity of Syt I.

## Materials and Methods

### Materials

Tris (hydroxymethyl) aminomethane (Tris), sodium dodecyl sulfate (SDS), Glycine, acrylamide (Acr), N, N′-methylenebisacrylamide (Bis), N, N, N′, N′-Tetramethylethylenediamine (TEMED) and ammonium peroxydisulfate (AP) were purchased from SERVA (Heidelberg, Germany). RC-DC™ Protein Assay Kit was from Bio-Rad (Hercules, CA, USA). Dithiothreitol (DTT), iodoacetamide (IAA), Triton X-100 and sequencing grade trypsin were from Sigma (St. Louis, MO, USA). Taq DNA polymerase, T4 ligase and all restriction enzymes used in cloning were from NEB (Ipswich, MA USA). *E. coli* Top10 was from Invitrogen (Paisley, UK). *E. coli* BL21-CodonPlus (DE3)-RIPL was from Stratagene (La Jolla, CA, USA). Expression vector pGEX-4T-1 and glutathione-sepharose beads were from Amersham Pharmacia Biotech (Uppsala, Sweden). Adult Sprague-Dawley rats (weighting 200–250 g) were purchased from the Center South University (Changsha, China). All the rats were allowed food and water ad libitum before being used in the experiments.

### Preparation of recombinant fusion proteins

The total RNA was extracted from rat brain tissues and the mRNA was purified with an E.Z.N.A^®^ Mag-Bind^®^ mRNA isolated Kit (Omega) according to the manufacturer’s instructions (Stamford, CT, USA). Reverse transcription of the mRNA into cDNA was performed using a PrimeScript™ 1st Strsnd cDNA Synthesis kit following the instructions of the manufacturer (Takara, Madison, WI, USA). The cDNA (P21707 (SYT1_RAT) Reviewed, UniProtKB/Swiss-Prot) was used as the template to obtain DNAs encoding Syt I C2A domain (residues 140–265) and C2B domain (residues 271–421) by PCR amplification with the forward primers containing EcoRI recognition site and the reverse primers containing XhoI recognition site. The forward primer and reverse primer for PCR amplification of C2A DNA were 5′-AGGAATTC GAGAAACTGGGAAAGCTC-3′and 5′-TCCTCGAGTCAAGCGCTCTGGAGATC-3′, respectively, and those for PCR amplification of C2B DNA were 5′-AAGAATTCGAGAAACTGGGTGACATC-3′and 5′-TCCTCGAGTCATTACTTCTTGACAGC-3′, respectively.

C2A and C2B GST fusion proteins were overexpressed in *E. coli* BL21-CodonPlus (DE3)-RIPL cells with glutathione S-transferase expression vector pGEX-4T-1. Glutathione-sepharose beads were used to affinity purify the expressed GST fusion proteins. Before being used, the beads were washed three time with 1× PBS buffer. After the beads and cell lysate were mixed and incubated overnight at 4 °C with continuous agitaion, centrifugation at 3,000 g for 5 min 4 °C was used to recover the beads, followed by washing three times with 1× PBS buffer. An aliquot of the beads were suspended in SDS loading buffer (0.5 M Tris–HCl, 4% SDS, 0.1 M DTT, 20% glycerol and trace of bromophenol blue, pH 6.8) and boiled for 10 min for SDS-PAGE analysis. The GST protein itself was also expressed and used as a control.

### GST pull-down

Rat brain was dissected and homogenized in a buffer (10 mM Hepes-NaOH, 150 mM NaCl, 1 µM pepstatin A, 2 µM leupeptin, 0.3 mM phenylmethylsulfonyl fluoride, pH7.4). After the homogenate was extracted for 1 h at 4 °C, insoluble materials were removed by centrifugation at 10,000 g for 30 min at 4 °C. The total extract was precleared by incubation for 3 h at 4 °C with glutathione-sepharose bead-bound GST protein. The precleared brain extract was recovered by centrifugation (3,000 g for 5 min) and separately incubated with glutathione-sepharose bead-bound C2A or C2B GST fusion proteins overnight at 4 °C. After incubation, the beads were recovered by centrifugation at 3,000 g for 5 min at 4 °C, and then washed twice with 500 µL RIPA buffer (50 mM Tris–HCl, 1% NP-40, 0.5% sodium deoxycholate, 0.1% SDS, 0.1mM PMSF, pH 7.4) containing 150 mM and 300 mM NaCl. The bound proteins were eluted from the beads with the addition of 2 × SDS loading buffer, followed by boiling for 10 min and centrifugation at 10,000 g for 5 min. The supernatant was collected and subjected to SDS-PAGE. The experiments were performed in triplicate.

### SDS-PAGE and in-gel digestion

The proteins interacting with C2 domains were separated by SDS-PAGE on 11.5% acrylamide separation gel and 4.8% stacking gel. After the electrophoresis was complete, the lane gel was cut into slices of about 2 mm wide. The slices were further cut in small pieces. The gel-bound proteins were detained, reduced with DTT (10 mM DTT in 25 mM NH_4_ HCO_3_) and alkylated with IAA (55 mM in 25 mM NH_4_ HCO_3_). After the gel pieces were washed and lyophilized, trypsin dissolved in 25 mM NH_4_ HCO_3_ and 10% ACN (1 µg/µL) was added and incubated at 37 °C for 16 h. The resulting peptides were extracted sequentially by 50% ACN/5% formic acid, 75% ACN/5% formic acid and 95% ACN/5% formic acid, each with sonication for 15 min. The extracts were combined and appropriately concentrated in a Speed-Vac and ready for CapLC-MS/MS analysis.

### CapLC-MS/MS and bioinformatics

The capillary LC-MS/MS analysis of the tryptic peptides was performed on an automated Agilent 1200 LC system (Agilent Technologies, Waldbronn, Germany) coupled with a 3D high-capacity ion trap mass spectrometer (HCTultra™, Bruker Daltonics, Bremen, Germany). Buffer A was 0.1% formic acid. Buffer B was ACN containing 0.1% formic acid. The peptides were eluted from the analytical capillary column (15 cm×180 µm, LC-Packings, Amsterdam, Netherlands) with the following gradient: 0–5% B over 5 min, 5–50% B over 60 min, 50–95% B over 10 min. The eluted peptides were directed into the mass spectrometer for MS/MS analysis. Peptides were analyzed in a positive mode and the five most abundant ions detected in each MS scan were selected for collision-induced dissociation (CID) using the data-dependent MS/MS mode over the m/z range of 200–2,000.

The acquired raw spectral data were processed and Mascot-compatible mgf files were created using DataAnalysis™ 3.4 software (Bruker Daltonics, Bremen, Germany). The SwissProt protein database (Taxonomy: Rattus) was used for protein identification. Search parameters were set as follows: enzyme, trypsin; allowance of up to one missed cleavage site; MS mass tolerance, 1.2 Da; MS/MS mass tolerance, 0.6 Da; fixed modification, carbamidomethylation (C); variable modification, oxidation (M). Proteins were identified on the basis of peptides whose ions scores exceeded the threshold, *P* < 0.05 , which indicated identification at the 95% confidence level. The relevant information on the identified proteins was retrieved from the related protein databases.

All the experimental procedures involving animals were approved by the Medical Ethics Committee of Hunan Normal University (approval number: 020). The scanned copy of the approval documentation was provided as a Supplemental File.

## Results and Discussion

### GST pull-down and SDS-PAGE

**Figure 1:**
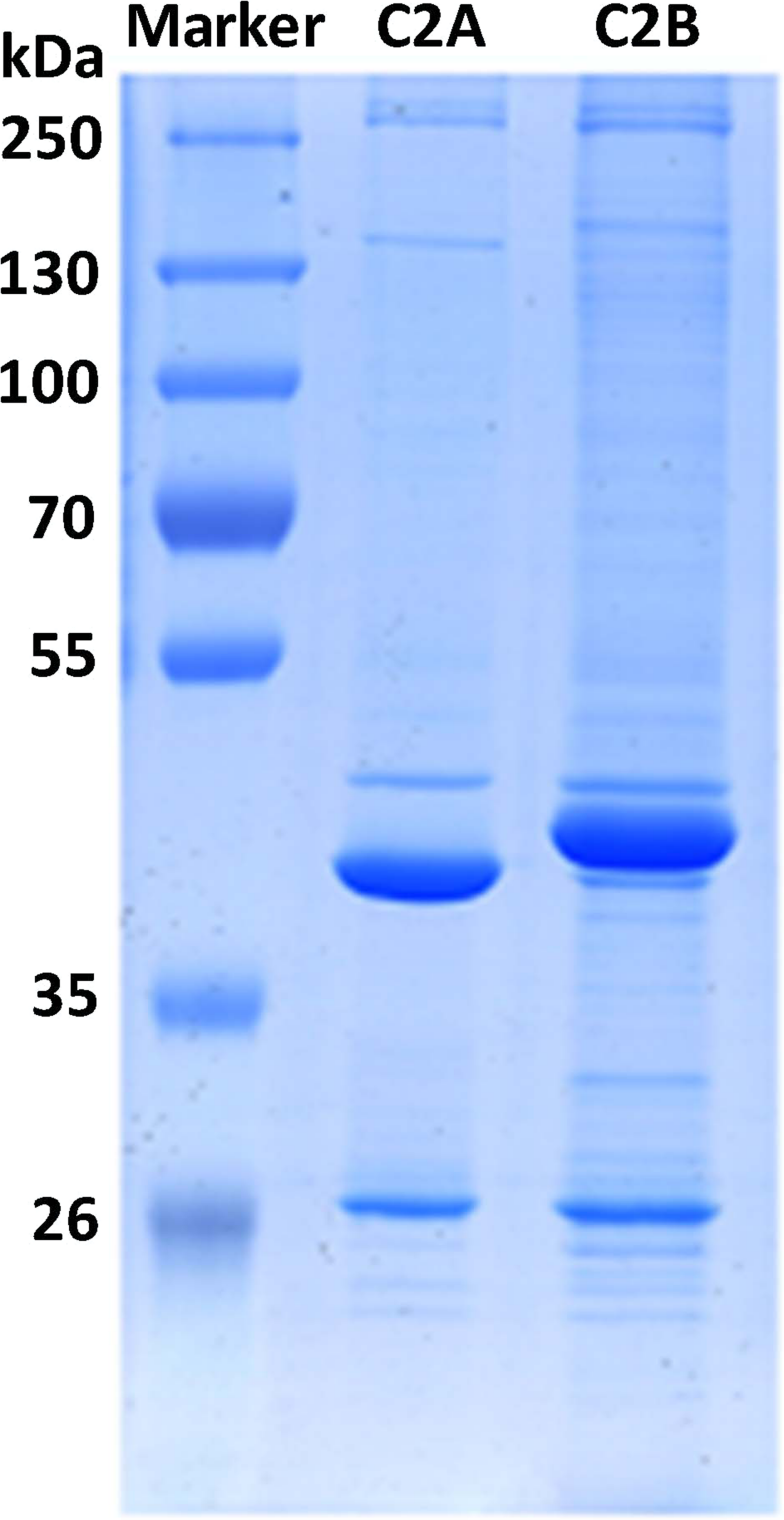
SDS-PAGE images of the proteins interacting with C2 domains.

In the present study, after the rat brain extract was extensively pretreated by incubation with glutathione-Sepharose bead-bound GST protein, the pre-cleared extract was divided into aliquots, followed by separate incubation with glutathione-Sepharose bead-bound C2A and C2B GST fusion proteins. For removing the non-specifically bound proteins, 150 mM and 300 mM NaCl solutions were used sequentially to wash the beads. The proteins that remained bound to the C2 domains were strongly resistant to the elution of the NaCl solutions, suggesting their particularly stable interactions. After the bound proteins were eluted down by the loading buffer and by boiling, SDS-PAGE was used to resolve the protein samples (Fig. 1). From the representative figure it can be seen that both C2A and C2B lanes exhibit a batch of protein bands. Comparatively, there are more bands in C2B lane, suggesting that C2B domain could bind even more proteins in the rat brain extract.

### Identification of the proteins interacting with C2 domains

Up to date, much effort has been made in the identification of the proteins that interact with the C2 domains of Syt I (Zhang et al., 1994; Schiavo et al., 1995; Sugita & Südhof, 2000; Honda et al., 2002; Chapman, 2008; Ciani et al., 2015; Gautam et al., 2015). However, to the best of our knowledge, there has not been a comprehensive study on such proteins. Contemporary proteomic technologies make it possible to identify these proteins sensitively, accurately and comprehensively. In the present study, we used the proteomic strategy in combination of other related techniques to systematically identify the proteins interacting with the C2 domains of Syt I. As a result, a total of 135 non-redundant proteins interacting with the C2 domains of Syt I were identified, of which 32 and 64 proteins only bound to C2A or C2B domains, respectively, and 39 proteins bound to both of the two domains (Table S1). The number of C2B domain-interacting proteins was twice that of C2A domain-interacting proteins. These data suggest that the two domains may mediate the same and different cellular processes via interacting with the same and different proteins, and that the C2B domain may play even more roles in the functioning of the Syt I, which may be partly due to the fact that the C2B domain is closer to the free C-terminus of Syt I than C2A and the less steric hindrance favors C2B domain interaction with various proteins. In addition, it is worth mentioning that literature survey indicated that the identified 135 proteins contain most of the proteins or their homologs that have been reported to bind to Syt I or its C2 domains (see below), demonstrating the reliability of the protein identification in our present work.

**Figure 2:**
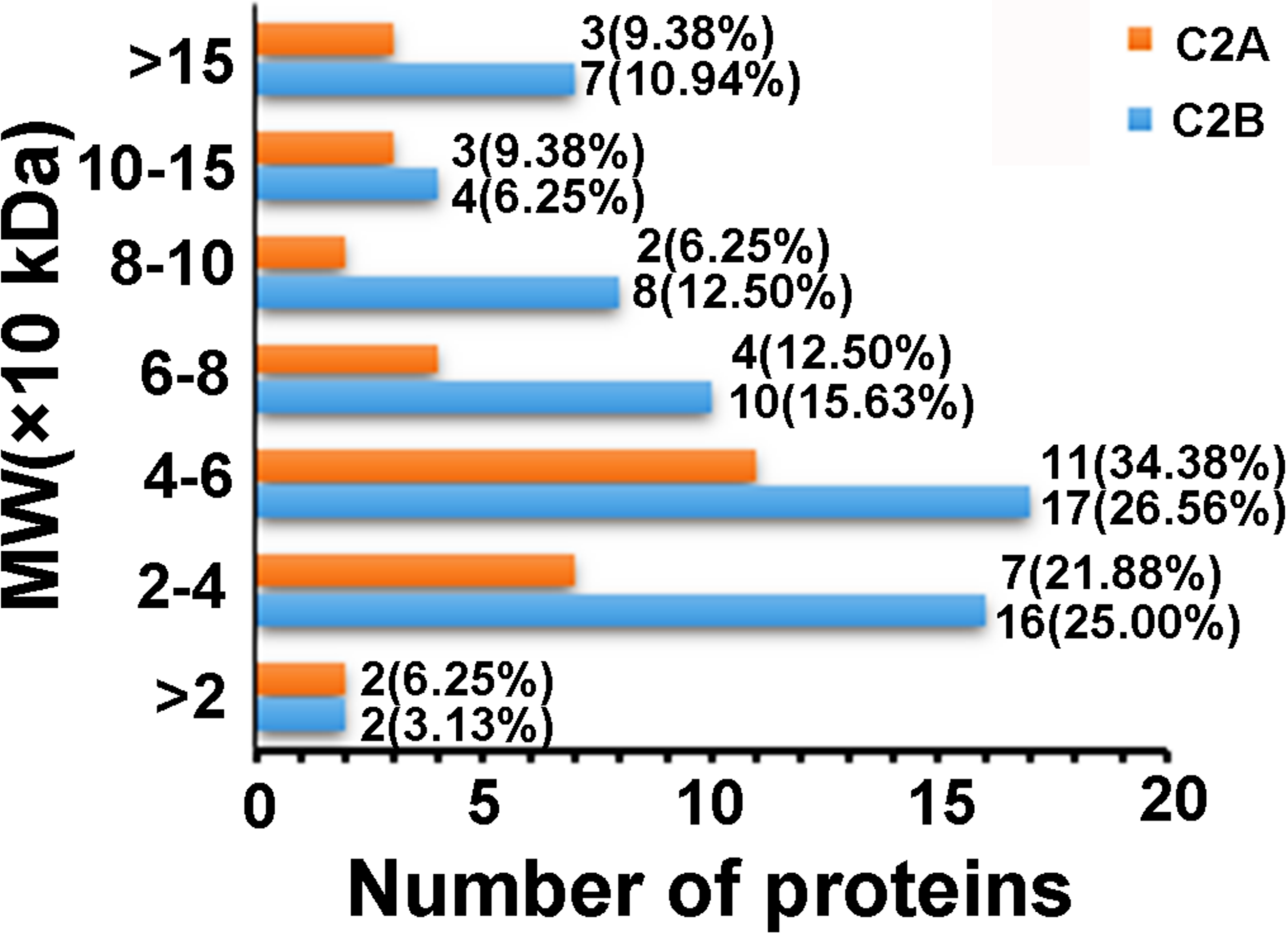
Distributions of the proteins that only interacted with C2A or C2B domains as a function of MW.

### MW and pI distributions

**Figure 3:**
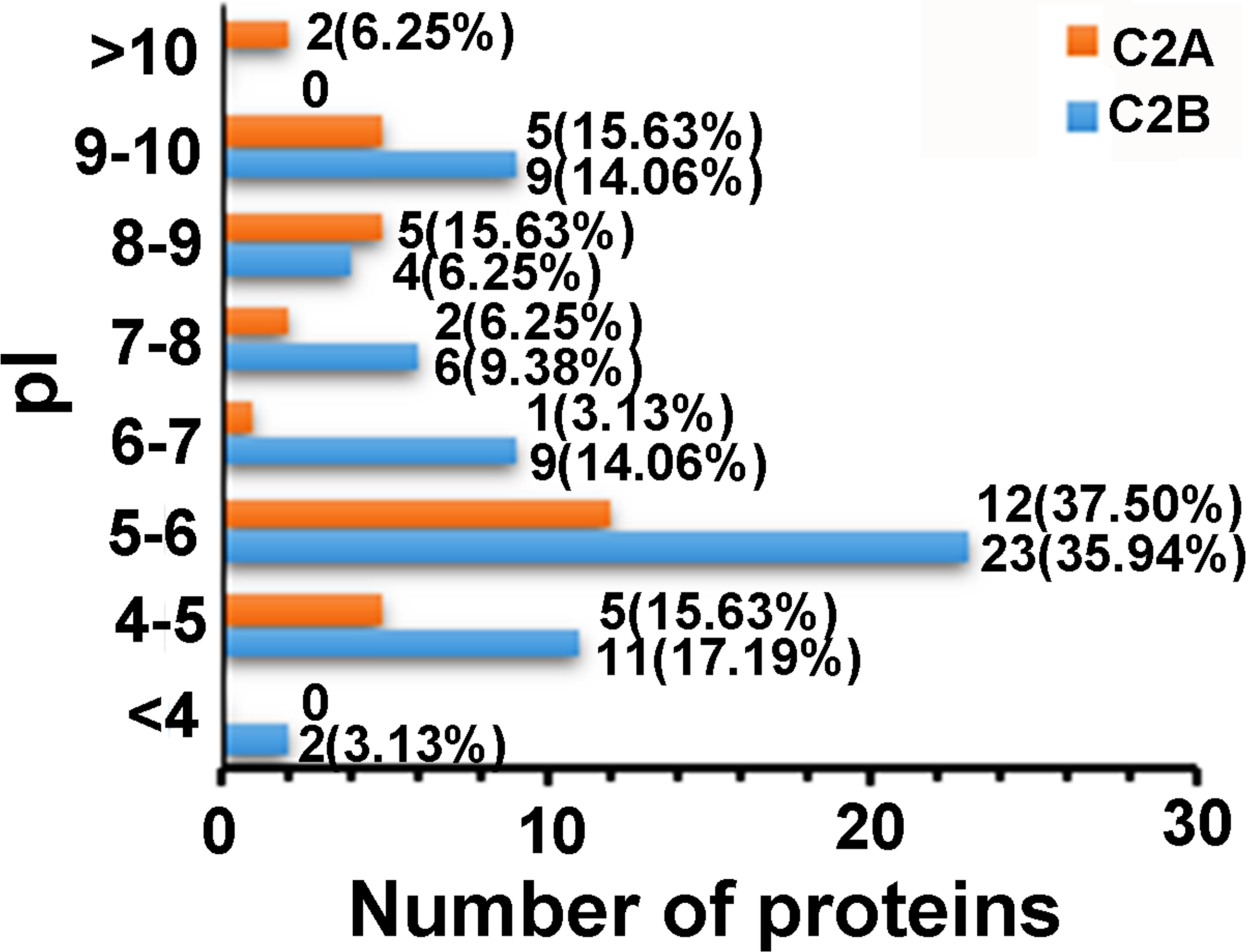
Distributions of the proteins that only interacted with C2A or C2B domains as a function of pI.

For further understanding of the C2 domain-interacting proteins, we first compared the distribution profiles of molecular weight (MW) and isoelectric point (pI) of the identified proteins. The MWs of the 32 proteins that only interacted with C2A domain were distributed in the range of about 8.25–580.50 kDa, with 56.26% of the proteins having MWs of 20–60 kDa, whereas those of the 64 proteins that only interacted with C2B domain in the range of about 1.09–347.48 kDa, with 51.56% of the proteins having MWs of 20–60 kDa. The MW distribution profiles of these two groups of proteins were similar to each other (Fig. 2). Figure 3 comparatively presents the pI distribution profiles of the proteins that only interacted with C2A or C2B domains, respectively. It can be seen that the pIs of the C2A domain-interacting proteins were distributed in the range of 4.46–11.25, whereas those of the C2B domain-interacting proteins in the range of 3.87–9.89. The pI distribution profiles of these two groups of C2 domain-interacting proteins were not obviously different, both of which had more than half of the proteins with pIs of 4–6. In addition, we also analyzed the MW and pI distribution profiles of the 39 proteins that interacted with both C2A and C2B domains. The results showed that the distribution profiles were similar to those of the proteins only interacting with C2A or C2B domains (Fig. S1). These results indicate that there are no obvious differences in the MW and pI distribution profiles of the proteins interacting with C2A or C2B domains.

**Figure 4:**
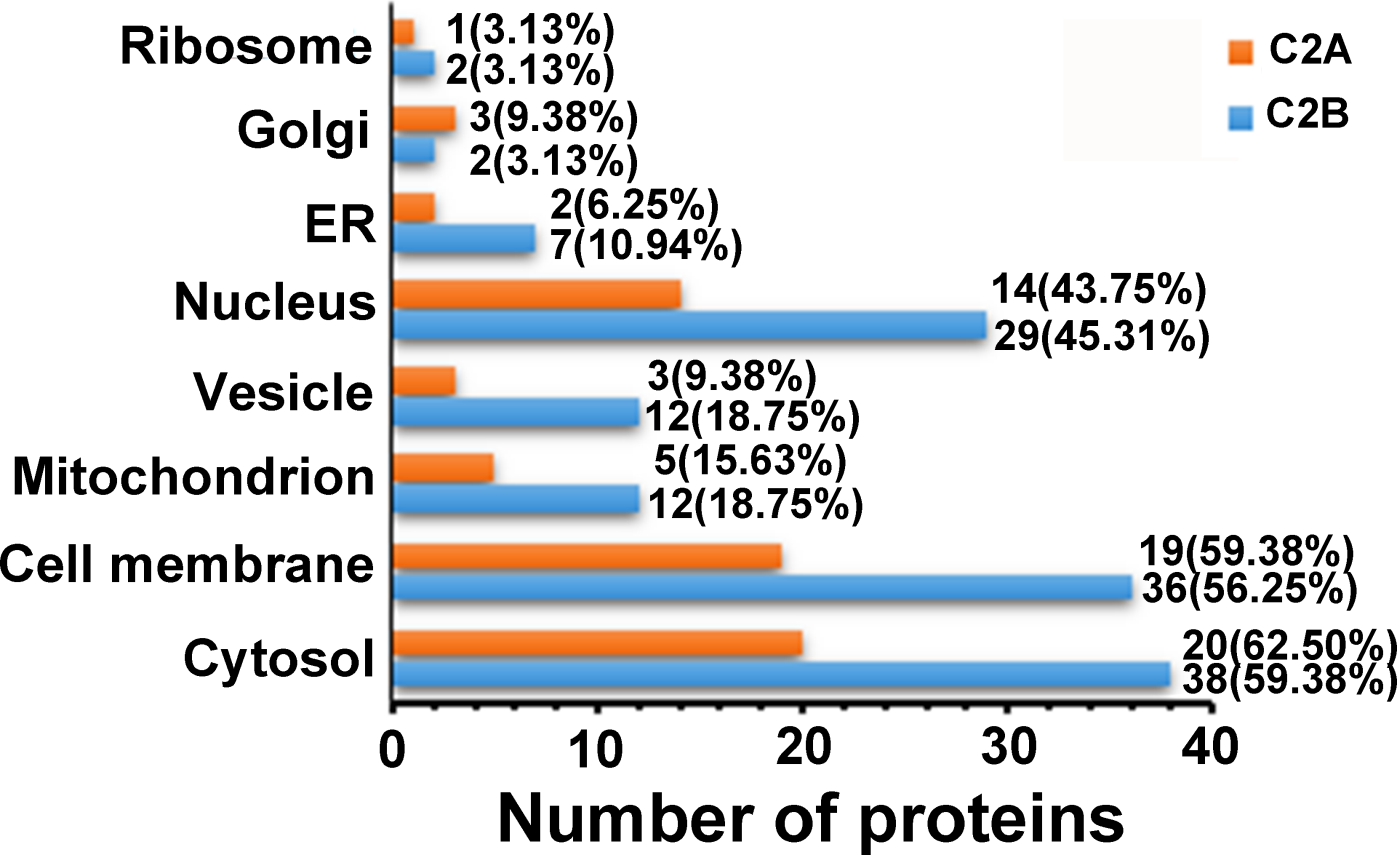
Subcellular localization of the proteins only interacting with C2A or C2B domains.

### Subcellular localization

In order to obtain further insight into the C2 domain-interacting proteins, we classified the identified proteins according to the universal GO cellular component annotation (Table S1). We analyzed the subcellular distribution of the identified C2 domain-interacting proteins based on eight subcellular localizations: cytosol, cell membrane, nucleus, vesicle, mitochondrion, endoplasmic reticulum, Golgi apparatus and ribosome. As shown in Fig. 4, for the 32 proteins only interacting with the C2A domain, cytosol and cell membrane are the main subcellular localizations, with 20 (accounting for 62.50%) and 19 (59.38%) proteins being distributed in the cytosol and cell membrane, respectively, followed by nucleus (14, 43.95%) and mitochondrion (5, 15.63%). Only less than 10% of the proteins have subcellular localizations in endoplasmic reticulum, Golgi apparatus or ribosome. Although the identified proteins only interacting with C2B are more than those only interacting with C2A (64 vs 32), their subcellular distribution profiles in the eight subcellular localizations are very similar. In addition, the subcellular localizations of the proteins that interacted with both C2A and C2B domains were also analyzed. The results showed that, of the 39 proteins interacting with the two C2 domains, 76.92% have their subcellular localizations in cytosol, 38.46% in nucleus, 35.90% in cell membrane, and 20.51% in mitochondrion (Table S1), indicating that most of the proteins interacting with both of the two C2 domains are distributed in the cytosol.

From the above analyses, we found that Syt I-interacting proteins are distributed in multiple subcellular compartments. It is worthy of noting that Syt I is generally considered to be localized in vesicular and cell membranes (Matthew, Tsavaler & Reichardt, 1981). However, in our present experiment some proteins in nucleus, mitochondrion, endoplasmic reticulum and Golgi apparatus, etc. were demonstrated to bind to C2 and/or C2B domains of Syt I. Obviously, such a phenomenon is partly due to the multiple subcellular localization of a protein; however, it suggests that in the cells Syt I might be transferred onto different organelles by for example membrane trafficking, which needs to be investigated further.

**Figure 5:**
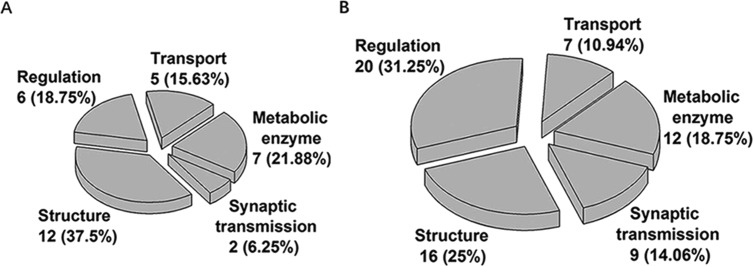
Functional classification of the proteins interacting only with the C2A (A) or C2B (B) domains, respectively.

### Functional survey

Through the GO annotation, we found that many C2 domain-interacting proteins have multiple functions and subcellular localizations. Furthermore, the subcellular localization of a protein may affect and determine its functions (Peng et al., 2015). Therefore, it is difficult to strictly classify these proteins based on their functions. However, in order to facilitate the analysis of biological functions of the identified proteins, we roughly classified these proteins into five groups according to the representative function of each protein: synaptic transmission, structure, regulation, transport and metabolic enzyme. As shown in Fig. 5 and Table S1, the differences between the proteins interacting with C2A or C2B domains were first reflected in the number of proteins classified into the various groups. Of the proteins interacting only with the C2A domain, the numbers of the proteins grouped in synaptic transmission, structure, regulation, transport and metabolic enzyme were 2, 12, 6, 5 and 7, respectively. Compared with C2A domain, C2B domain could bind to more proteins particularly those involved in synaptic transmission (9 vs 2) and regulation (20 vs 6). These data demonstrate that the C2B domain is even more involved in various cellular processes especially synaptic transmission and metabolic regulation than C2A domain. In addition, of the 39 proteins that interacted with both C2A and C2B domains, 26 proteins (accounting for 66.67%) had structural molecular activity, suggesting that structural proteins are more inclined to bind to both of the two C2 domains.

**Table 1:** The C2 domain-interacting proteins closely related to synaptic transmission.

ID	Protein name	Main function	Bind to C2A/C2B
PCLO_RAT	Protein piccolo	Organization of synaptic active zones; Synaptic vesicle trafficking.	C2A
SGIP1_RAT	SH3-containing GRB2-like protein 3-interacting protein 1	Clathrin-mediated endocytosis and synaptic vesicle recycling.	C2A
EAA1_RAT	Excitatory amino acid transporter 1	Terminating the postsynaptic action of glutamate.	C2B
AP2A2_RAT	AP-2 complex subunit alpha-2	Recycling of synaptic vesicle membranes from the presynaptic surface; Scaffolding platform for endocytic accessory proteins.	C2B
AP2M1_RAT	AP-2 complex subunit mu	Recycling of synaptic vesicle membranes from the presynaptic surface; Binds to transmembrane cargo proteins.	C2B
SNP25_RAT	Synaptosomal-associated protein 25	Vesicle docking and membrane fusion.	C2B
NSF_RAT	Vesicle-fusing ATPase	Vesicle-mediated transport; Neurotransmitter secretion.	C2B
KPCG_RAT	Protein kinase C gamma type	Protein phosphorylation; Mediation of synaptic function.	C2B
STX1B_RAT	Syntaxin-1B	Docking of synaptic vesicles at presynaptic active zones.	C2B
NRX1A_RAT	Neurexin-1-alpha	Synapse assembly; Vesicle docking; Neuromuscular process controlling balance.	C2B
MYO5A_RAT	Unconventional myosin-Va	SNARE binding; Syntaxin-1 binding; Transport of vesicles to the plasma membrane.	C2B
CAC1B_RAT	Voltage-dependent N-type calcium channel subunit alpha-1B	Involved in a variety of calcium-dependent processes, including neurotransmitter release.	C2A & C2B
KCC2A_RAT	Calcium/calmodulin-dependent Protein kinase type II subunit alpha	Regulation of neuronal synaptic plasticity and neurotransmitter secretion.	C2A & C2B
GLNA_RAT	Glutamine synthetase	Positive regulation of synaptic transmission.	C2A & C2B

#### Proteins closely related to synaptic transmission

In the present study, we classified 14 identified proteins or subunits that are closely related to synaptic transmission into this group (Table 1), though there are other identified proteins being related to synaptic transmission (see below). These proteins participate in synaptic transmission by transporting ions, organizing synaptic active zones, recycling synaptic vesicle membranes, mediating vesicle docking and membrane fusion, and/or regulating synaptic transmission. It can been seen from the Table 1 that C2B domain could bind to even more proteins involved in synaptic transmission than C2A, demonstrating that C2B domain of Syt I plays even more important roles in the cellular process. Of the 14 identified proteins or subunits, six have already been reported to bind to Syt I: AP-2 complex subunit alpha-2 (Zhang et al., 1994; Haucke et al., 2000), AP-2 complex subunit mu (Zhang et al., 1994; Haucke et al., 2000), synaptosomal-associated protein 25 (Gerona et al., 2000; Rickman et al., 2004), syntaxin-1B (Rickman et al., 2004; Xie et al., 2014), neurexin-1-alpha (Perin, 1994), and voltage-dependent N-type calcium channel (Sheng, Yokoyama & Catterall, 1997). The rest proteins were newly identified. These findings provide new clues for further investigation of the Syt I-mediated synaptic transmission.

#### Proteins with structural molecular activity

In this work, a considerable proportion of the proteins that were identified to bind to the C2 domains have typical structural molecular activity, and thus were classified into the group of “structure,” though they also have other biofunctions. Most of them are cytoskeletal proteins involved in the synaptic vesicle cycle and distribution, such as keratin, tubulin and actin (Table S1), of which only tubulin had been proven to directly bind to Syt I (Honda et al., 2002) and actin had been reported to bind to the C2A domain of Syt I (Sugita & Südhof, 2000). Of the proteins that were identified to interact with C2A and/or C2 domains, keratin cytoskeleton proteins account for a certain proportion. This result suggests that, like tubulins (Honda et al., 2002) and actins (Sugita & Südhof, 2000), keratin proteins also play important roles in Syt I-mediated cellular processes. It is worth mentioning that most of the identified cytoskeletal as well as cytoskeleton-associated proteins were shown to bind to both C2A and C2B domains, suggesting that the vesicle transport and distribution are heavily dependent on the synergistic actions of the two C2 domains and these structural proteins. Besides, our present study demonstrated that C2 domains of Syt I interacted not only with the above-mentioned cytoskeletal as well as cytoskeleton-associated proteins, but also with several structural membrane proteins, including myelin proteolipid protein, myelin basic protein S, glypican-1, syndecan-3, syndecan-4, and band 4.1-like protein 1, etc. All of these membrane proteins were newly found to interact with Syt I. These results indicate that the biological roles of Syt I involve the C2 domain interaction with both cytoplasmic and membrane proteins.

**Figure 6:**
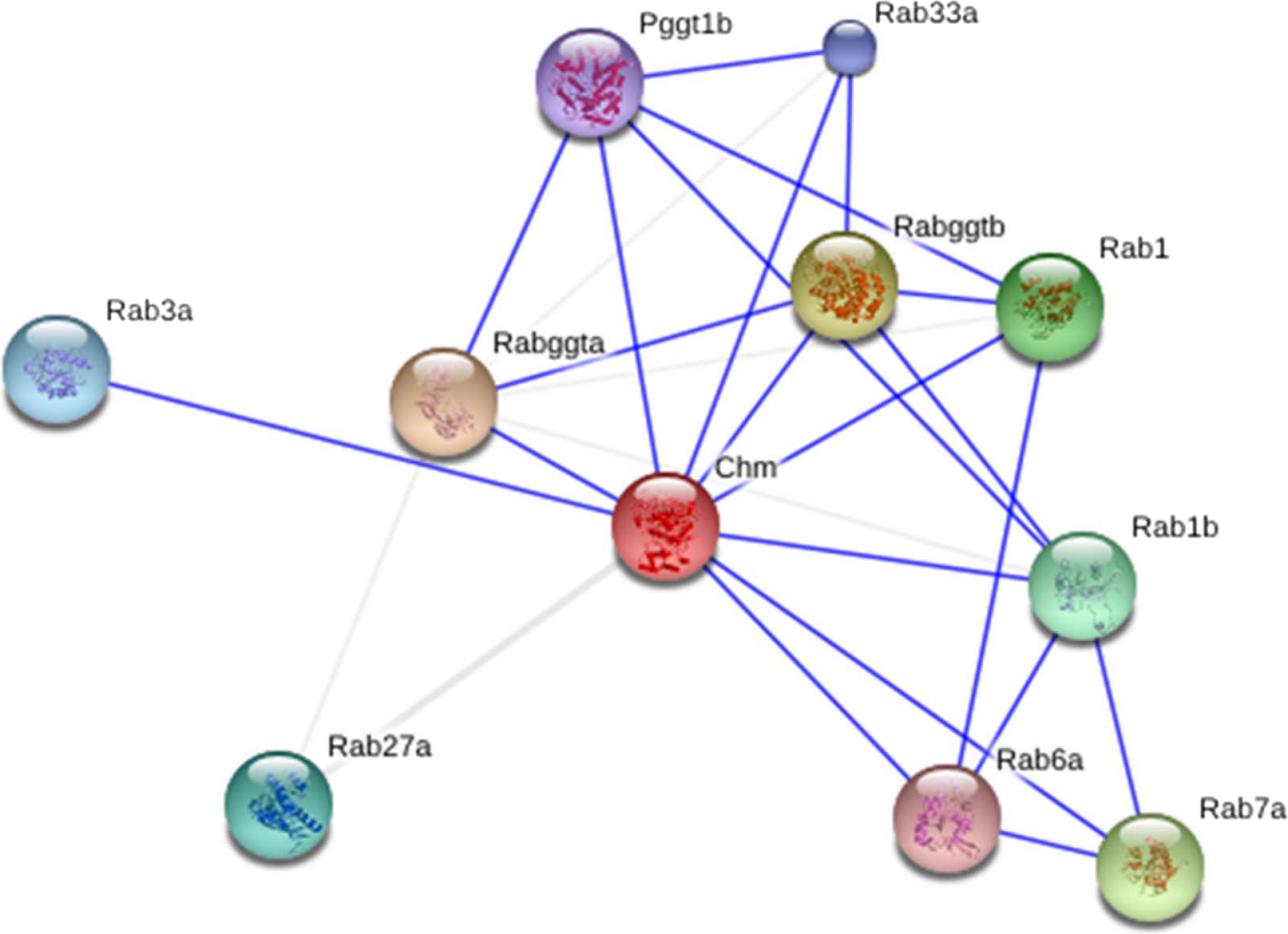
Interactions of Rab proteins geranylgeranyltransferase component A 1 (Chm) with Rab proteins.

#### Regulatory proteins

Of the 135 identified proteins there are a batch of proteins with regulatory function. Due to their multiple functions, a part of them were classified into the groups of “structural proteins” as well as other groups, but most of them were put into the group of “Regulatory proteins” (Table S1). Literature survey showed that in this group only casein kinase II subunits alpha and beta have already been unambiguously identified to interact with Syt I. Syt I has been proven to be one of the major substrates in brain for casein kinase II, which phosphorylates Syt I at a single threonine. The possible roles of the phosphorylation of Syt I could be modulation of its Ca^2+^ binding properties and interactions with other molecules, thus exerting regulatory effects on the synaptic exocytosis (Davletov et al., 1993). In addition, more than two dozen proteins were newly identified to interact with the C2 domains of the Sty I, including Rab proteins geranylgeranyltransferase component A 1, protein S100-A5, nucleobindin-1, etc. (Table S1), suggesting that Syt I may regulate multiple metabolic pathways via those interacting proteins. For example, of the regulatory proteins identified, Rab proteins geranylgeranyltransferase component A 1 is an important regulatory protein. It is a substrate-binding subunit of the Rab geranylgeranyltransferase (GGTase) complex, binding unprenylated Rab3 proteins, presenting it to the catalytic Rab GGTase dimer, and remaining bound to it after the geranylgeranyl transfer reaction (Fig. 6). Thus, it is speculated that Syt I may regulate the function of Rab3, also a key protein in the regulation of synaptic vesicle exocytosis (Andres et al., 1993; Geppert & Südhof, 1998). In addition, several non-enzyme regulatory proteins are found to be functionally related to Ca^2+^ , including Protein S100-A5 and Nucleobindin-1, suggesting that they may play some cooperative roles with Syt I in the regulation of Ca^2+^ -mediated cellular processes. Lastly, it is worth pointing out that, among the identified Syt I-binding proteins, a batch of proteins may function in the regulation of transcription and translation. For example, protein SET is a multitasking protein, involved in transcription, nucleosome assembly and histone chaperoning and apoptosis. Whether and how the Syt I is transferred onto nucleus to regulate transcription by for example membrane trafficking needs further investigation.

#### Transport proteins

A total of 13 proteins were classified into this group, all of which were newly identified to bind to Syt I. They are all localized in cell membrane, vesicular membrane, mitochondrion inner membrane and/or nuclear membrane. These proteins facilitate the transport of various substances, such as phosphate by sodium-dependent phosphate transport protein 2A, coenzyme A by mitochondrial coenzyme A transporter SLC25A42, nucleotide analogs by ADP/ATP translocase 1 and multidrug resistance-associated protein 5 (Wijnholds et al., 2000), 2-oxoglutarate by mitochondrial 2-oxoglutarate/malate carrier protein, and importin subunit alpha-6 (Plant et al., 2006). Besides, several ion channel proteins/enzymes were demonstrated to bind to the C2 domains, including sodium/potassium-transporting ATPase, potassium voltage-gated channel subfamily C member 1, potassium-transporting ATPase alpha chain 1, calcium-transporting ATPase type 2C member 2. These ion channel proteins mediate the transmembrane transport of ions. If localized in synapses, they may participate in the regulation of synaptic transmission. Taken together, these results suggest that Syt I, as a membrane-bound protein, mediate the transmembrane transport of multiple substances by interacting with other membrane proteins, thereby affecting the membrane potential as well as substance and energy metabolism in cells.

#### Metabolic enzymes

In the current study, due to the multifunction of a protein, only 26 C2 domain-interacting proteins/subunits were classified into the group of “Metabolic enzyme,” though many more identified proteins have catalytic activity. All these enzymes were newly identified to interact with Syt I. Analysis of the subcellular localizations of the proteins in this group discovered that at least 15 of the 26 “Metabolic enzymes” have mitochondrial inner membrane or matrix localization, including those that are involved in oxidative phosphorylation, such as ATP synthase subunits (alpha, beta, gamma, b, e, etc.), NADH-ubiquinone oxidoreductase 75 kDa subunit, succinate dehydrogenase [ubiquinone] flavoprotein subunit, cytochrome b-c1 complex subunit 2, etc. These results suggest that Syt I might mediate the functions of mitochondria to a considerable extent by interacting with a series of mitochondrial proteins. Besides, Syt I can interact with multiple kinds of other metabolic enzymes, such as E3 ubiquitin-protein ligase UBR4, aconitate hydratase, phosphatidylinositol 5-phosphate 4-kinase type-2 beta, demonstrating that Syt I may extensively mediate the metabolic processes of proteins, carbohydrates, lipids, etc. in cells.

## Conclusions

In this work, we made a global and comprehensive analysis of the interaction proteins of the C2 domains of Syt I. The results demonstrate that Syt I may exert impacts by interacting with other proteins on multiple physiological and biochemical processes in cells, including vesicular membrane trafficking, synaptic transmission, metabolic regulation, catalysis, transmembrane transport and structure formation, etc., demonstrating that the functional diversity of Syt I is higher than previously expected. Its two domains may mediate the same and different cellular processes. Comparatively, C2B domain could bind many more proteins than C2A, particularly the proteins involved in synaptic transmission and metabolic regulation, indicating that C2B domain may play even more important roles in the functioning of the Syt I.

##  Supplemental Information

10.7717/peerj.2973/supp-1 Figure S1

10.7717/peerj.2973/supp-2 Table S1 Information on the proteins interacting with C2 domains of Syt I

## List of abbreviations

LC-MS/MS: liquid chromatography-tandem mass spectrometry
SDS-PAGE: sodium dodecyl sulfate polyacrylamide gel electrophoresis
SNARE: soluble N-ethylmaleimide-sensitive factor attachment proteins receptor
SNAP-25: Synaptosomal-associated protein 25
Syt I: synaptotagmin I
C2A: C2A domain of Syt I
C2B: C2B domain of Syt I.

## Additional Information and Declarations

### Data Availability

The following information was supplied regarding data availability: The raw data has been supplied as a Supplemental File.
