# Supplementary material for: Pull-down combined with proteomic strategy reveals functional diversity of synaptotagmin I
Source: PeerJ. 2017 Feb 8;5:e2973. doi: 10.7717/peerj.2973 (PMC5301975; doi:10.7717/peerj.2973)
Supplement: Figure S1 [file peerj-05-2973-s001.pdf]

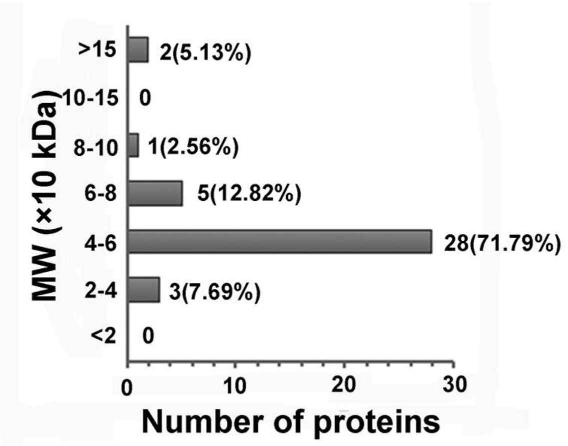

Supplementary Figure 1 Distributions of the proteins that interacted with both C2A and C2B domains as a function of MW.

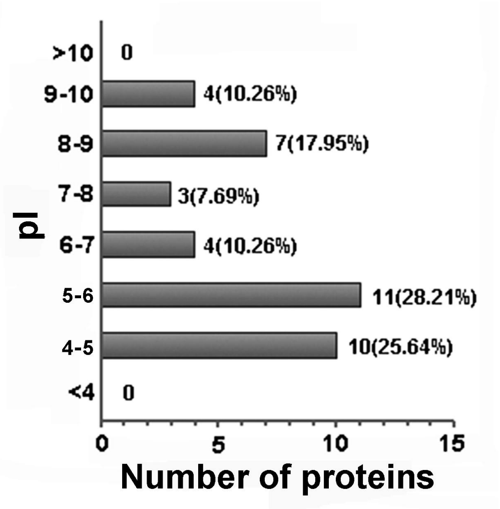

Supplementary Figure 2 Distributions of the proteins that interacted with both C2A and C2B domains as a function of pI.
